# Topical ocular dexamethasone decreases intraocular pressure and body weight in rats

**DOI:** 10.1186/s12952-016-0048-x

**Published:** 2016-03-12

**Authors:** Kota Sato, Koji M. Nishiguchi, Kazuichi Maruyama, Satoru Moritoh, Kosuke Fujita, Yoshikazu Ikuta, Hitoshi Kasai, Toru Nakazawa

**Affiliations:** Department of Ophthalmology, Tohoku University Graduate School of Medicine, 1-1, Seiryo, Aoba, Sendai, Miyagi 980-8574 Japan; Department of Advanced Ophthalmic Medicine, Tohoku University Graduate School of Medicine, Miyagi, Japan; Department of Retinal Disease Control, Tohoku University Graduate School of Medicine, Miyagi, Japan; Institute of Multidisciplinary Research for Advanced Materials, Tohoku University, Miyagi, Japan

**Keywords:** Glucocorticoide, Glaucoma, Intraocular pressure, Body weight, ER stress, Blood tests

## Abstract

**Background:**

Recently, topical dexamethasone-induced ocular hypertension and a consequent loss of retinal ganglion cells (RGCs) have been described in mice. This has been proposed as a model of steroid-induced glaucoma. In this study, we set up and evaluated a similar model in rats.

**Results:**

Ten-week old Sprague Dawley (SD) rats (*N* = 12) were used to evaluate the effect of topical 0.1 % dexamethasone (50 μl) administered 3 times daily for 4 weeks. Sodium chloride (0.9 %) was used in another group of rats (*N* = 12) that served as the controls. After 1 week, we observed a progressive decrease in body weight in the dexamethasone-treated rats compared both to the pre-treatment baseline and the vehicle-treated rats. In contrast to earlier work that showed elevated Intraocular pressure (IOP) following dexamethasone instillation in mice, IOP in the rats unexpectedly fell to 11.3 ± 1.3 mmHg in the treated eyes, compared to 14.8 ± 2.4 mmHg in the untreated eyes, after 3 weeks of topical dexamethasone (*P* = 0.032). Blood tests performed after 4 weeks of treatment showed a 3.3-fold increase in both plasma cholesterol (*P* < 0.001) and alanine transaminase (*P* = 0.019) in the dexamethasone-treated rats compared to the control rats. Meanwhile, topical steroid did not induce changes in either plasma blood glucose or glycated hemoglobin (HbA1c). We also did not detect changes in the expression of RGC markers (with real-time PCR) following the treatment.

**Conclusions:**

In contrast to mice, which previously showed increased IOP following the topical administration of dexamethasone, the rats displayed a paradoxical reduction in IOP following a similar treatment. This was accompanied by a loss of body weight without affecting the level of blood glucose.

**Electronic supplementary material:**

The online version of this article (doi:10.1186/s12952-016-0048-x) contains supplementary material, which is available to authorized users.

## Background

Glaucoma is a common cause of visual impairment, affecting about 70 million people worldwide [1, 2]. The condition is characterized by a selective loss of the retinal ganglion cells (RGCs) and their nerve fibers, resulting in a progressive narrowing of the visual field [3]. High intraocular pressure (IOP) is recognized as one of the risk factors for glaucoma [4]. Available treatments for glaucoma thus mostly rely on the pharmacological and/or surgical reduction of IOP.

In the classical model of steroid action, steroid molecules bind with steroid receptors and modulate transcription of various genes [5, 6]. Glucocorticoid, which is a class of steroid hormone, has anti-inflammatory effects, and is used in the clinical treatment of patients with diseases such as autoimmune disorders, allergies, and intraocular inflammation including uveitis and optic neuritis [7–9]. On the other hand, glucocorticoid treatment also has numbers of adverse effects, such as gain of weight, increased blood glucose, triglyceride, and cholesterol as well as elevated blood pressure [10, 11].

Some cases of elevated IOP occur as the off-target result of systemic or topical glucocorticoid therapy when patients are treated for various conditions unrelated to glaucoma [12, 13]. If not diagnosed and treated promptly, this IOP elevation can eventually induce a loss of RGCs and result in the development of steroid-induced glaucoma. This problem is not uncommon, as the topical administration of glucocorticoid, such as dexamethasone or betamethasone, can elevate IOP in approximately 30–40 % of the general population [12–17]. However, the pathological mechanisms of steroid-induced glaucoma are poorly understood, because an animal model has not been available to emulate this condition. Recently, however, a model of steroid-induced glaucoma accompanied by ocular hypertension and a subsequent loss of RGCs was established, in which topical treatment with dexamethasone was used for 6 weeks in C57BL/6J mice [18]. Detailed analysis of this model revealed that endoplasmic reticulum (ER) stress played a critical role in its pathology, suggesting that the suppression of ER stress is a promising approach to treat steroid-induced glaucoma.

Research into the pathological mechanisms of ocular disease can gain a few advantages by using rats rather than mice. One of the clearest is that IOP can be reliably measured in rats. Applying a tonometer to the center of a mouse cornea, which measures ~ 3.5 mm in diameter, is technically far more complicated and more prone to variation than the same procedure in rats, which have a substantially bigger cornea (~7.0 mm in diameter). Another advantage is that, in general, rats are more tolerant of behavioral tests than mice. This is important, as in vivo functional assessment of the RGCs is difficult, making visually cued behavioral testing a particularly informative way of evaluating the visual status of animals with RGC loss.

In this study, we attempted to develop a model of steroid-induced ocular hypertension and glaucoma in rats by applying a treatment regimen similar to that reported to induce this pathology in mice. Surprisingly, we found that topical steroid administration reduced IOP in rats, a result that was contradictory to previous findings in mice.

## Methods

### Animals

Ten-week-old male Sprague Dawley (SD) rats were obtained from SLC (Shizuoka, Japan). All animals were maintained and handled in accordance with the Association for Research in Vision and Ophthalmology (ARVO) Statement for Use of Animals in Ophthalmic Vision Research and the Tohoku University Guidelines for Animal Research. All experimental procedures were conducted after approval by the ethics committee for animal experiments at the Tohoku University Graduate School of Medicine.

### Treatment with topical ocular dexamethasone

Dexamethasone 21-phosphate disodium (Sigma, St. Louis, Mo, USA) was dissolved in saline (Otsuka Pharmaceutical, Japan) to make a 0.1 % solution. Sodium chloride (0.9 %; saline) served as the vehicle control. Either dexamethasone or vehicle was applied topically to the right eyes (50 μl/eye) of the rats (*N* = 12 per group) 3 times daily. The amount of steroid used was calculated based on previous work performed with mice [18]. The initial plan to administer the eye drops for 6 weeks was altered to 4 weeks following the unexpected reduction in IOP. The left eyes of the rats were not treated.

### Measurement of IOP and body weight

After the rats were anesthetized with isoflurane vaporized with NARCOBIT-E (Natsume Seisakusho, Tokyo, Japan), IOP was measured between 9 AM and 2 PM by applying a rebound tonometer (Tonorab; TioLat, Helsinki, Finland) to the center of the cornea. Body weight was measured immediately after IOP measurement. This process was repeated every week.

### Biochemical analysis of peripheral blood

The rats were deeply anesthetized with the intramuscular administration of a mixture of ketamine (500 mg/kg) and xylazine (45 mg/kg). Blood samples were then gently extracted from the heart after opening the thorax. The blood samples were centrifuged and the supernatant was collected as plasma, which was then sent to SRL, Inc. (Tokyo, Japan) for biochemical analysis. A small amount of whole blood was also collected in collection tubes containing sodium fluoride to measure glycated hemoglobin (HbA1c).

### Western blotting

The anterior segment was collected by enucleating the eye and dissecting and removing the posterior segment (containing the retina, sclera, and choroid) and lens from the rat eyes [18] and prepared for SDS-PAGE, as previously described [19]. Ten micrograms of protein per lane were loaded onto 10 % polyacrylamide gel followed by electrophoresis and size separation of the proteins. The proteins were then transferred to a PVDF membrane, as previously described [20]. The membranes were incubated with rabbit anti-CHOP (GADD153) antibody (sc-575, 1:1000, Santa Cruz Biotechnology, Delaware, CA, USA) or rabbit anti-ATF4 (CREB2) antibody (sc-200, 1:1000, Santa Cruz) as primary antibodies overnight at 4 °C. After washing with Tween-PBS, HRP-conjugated goat anti-rabbit antibody (Sigma) was incubated as a secondary antibody at room temperature for 1 h. Immunoblots were visualized with ECL prime detection reagents (GE Healthcare, Piscataway, NJ, USA) and immunosignal bands were captured with ChemiDoc XRS (Bio-Rad, Hercules, CA, USA). To detect beta-actin as an internal control, the membrane was reblotted with Restore Western Blot Stripping Buffer (Thermo Scientific, Hudson, NH, USA). Mouse anti-beta-actin antibody was used as the primary antibody. The expression of CHOP and ATF4 was induced by incubating RGC5 cells with 4 μg/ml of tunicamycin (Wako Pure Chemical Industries, Osaka, Japan) in a humidified atmosphere of 5 % CO_2_ and 95 % air at 37 °C overnight.

### Quantitative reverse-transcription PCR

Total RNA collected from the rat retinas was isolated with an miRNeasy Mini Kit (Qiagen, Hilden, Germany), according to the manufacturer’s instructions. Total RNA (1 μg each) was used to generate first strand cDNA using a SuperScript III First-Strand Synthesis SuperMix for qRT-PCR (Life Technologies, Inc., MD, USA). Quantitative PCR (qPCR) was performed using a 7500 fast real-time PCR system (Applied Biosystems, Foster City, CA, USA) and amplified with TaqMan Fast Universal PCR Master Mix (2X), No AmpErase UNG (Applied Biosystems). The reaction was performed under the following condition: 95 °C for 20 s, 40 cycles of 95 °C for 3 s and 60 °C for 20 s. To amplify and detect the signal, predesigned TaqMan probes (Life Technologies, Inc.) were used, as follows: Thy1 (Rn00562048_m1), Nefh (Rn00709325_m1), Pou4f1 (Rn01753495_m1), Pou4f2 (Rn01431271_g1), Pou4f3 (Rn00454761_g1), and Gapdh (Rn01462662_g1). Each starting template was normalized to Gapdh mRNA. Relative mRNA levels were calculated with the delta-delta Ct method.

## Results

### Topical ocular dexamethasone decreased intraocular pressure and body weight in the rats

The purpose of this study was to establish a rat model of steroid-induced glaucoma by applying a protocol similar to that used to elevate IOP in mice [18]. Therefore, we initially planned to instill the dose-adjusted dexamethasone eye drops 3 times daily for the period of 6 weeks as described in the mouse protocol [18].

**Fig. 1:**
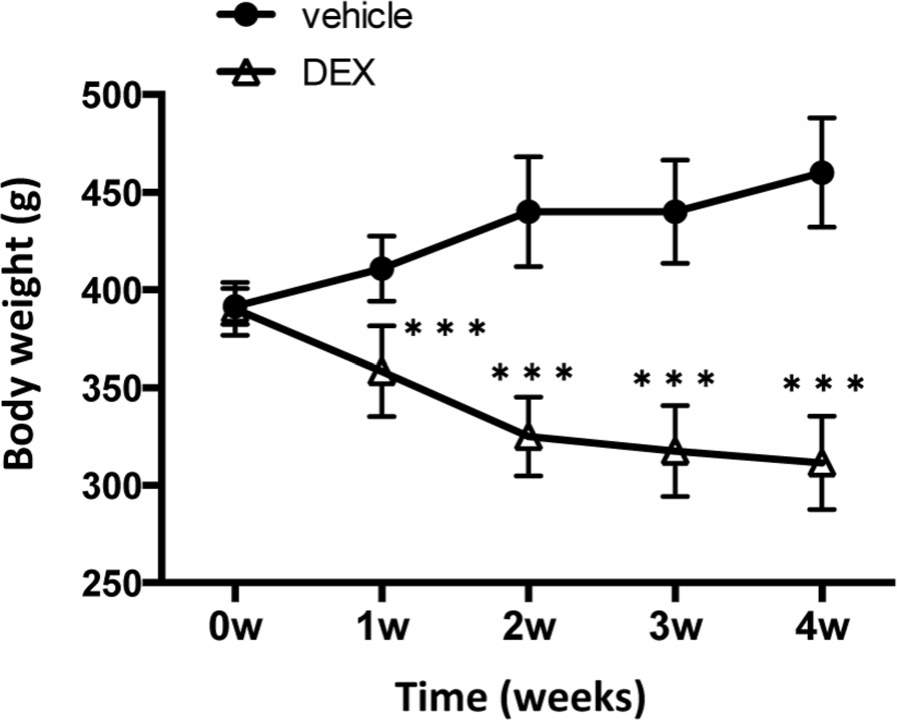
Loss of body weight after topical dexamethasone treatment. The body weight of vehicle-treated (0.9 % sodium chloride) and DEX-treated (0.1 % dexamethasone) rats was measured weekly, after IOP measurements. The graph shows the mean ± standard deviation of the rats’ body weight (*N* = 12). ^***^ *P* < 0.001, unpaired t-test

**Fig. 2:**
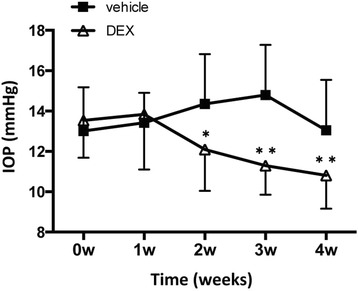
IOP reduction after topical dexamethasone treatment. Topical ocular vehicle or DEX was administered 3 times daily for up to 4 weeks. IOP measurements from vehicle-treated and 0.1 % dexamethasone-treated rats are shown. Values represent the mean ± standard deviatio of the mean (*N* = 12). ^*^ *P* < 0.05, ^**^ *P* < 0.01, unpaired t-test

However, after 1 week of topical ocular treatment with 0.1 % dexamethasone, we noted an unexpected, significant decline in the body weight of the dexamethasone-treated rats (358.3 ± 23.3 g; mean ± standard deviation) compared to the saline-treated rats (410.8 ± 16.8 g; mean ± standard deviation; *P* = 0.0004; Fig. 1). The body weight of the steroid-treated rats continued to show a steady decline in the following weeks, and had decreased to ~66.6 % of that of the saline-treated group after treatment for 4 weeks. Even more surprisingly, we observed that this decline in body weight was accompanied by a reduction in IOP starting after 2 weeks of treatment. IOP was significantly lower (falling to ~76.3 % of that of the saline-treated rats) in the steroid-treated eyes (11.3 ± 1.4 mmHg) than in the control eyes (14.8 ± 2.4 mmHg; *P* = 0.0032; Fig. 2) after 3 weeks of administration. In addition, IOP in the untreated contralateral left eyes also decreased after topical dexamethasone treatment in rats (Additional file 1: Figure S1), leading us to speculate that the IOP reduction in the DEX-treated rats was due to a systemic influence, not an effect specific to the eyes. Similar results were obtained after 4 weeks of steroid instillation. At this point, we decided to discontinue the study as it was clear that the rats were responding very differently than mice, and that completing the initially planned 6 weeks of treatment would not bring us closer to our goal of establishing a rat model of ocular hypertension and steroid-induced glaucoma.

Discontinuing the experiment was also the most appropriate response considering the ethical norms of our institution’s guidelines for animal research.

### Topical ocular dexamethasone increased plasma cholesterol and alanine transaminase without affecting blood glucose

In order to better understand the medical phenomenon underlying the decline in body weight and the reduction in IOP caused by the steroid eye drops, we collected plasma from the rats after 4 weeks of treatment and analyzed its biochemical properties. The results showed that cholesterol and alanine transaminase (ALT) levels were dramatically higher (both by ~3.3-fold) in the steroid-treated rats than the saline-treated rats (Table 1). On the other hand, the creatinine level significantly decreased in the steroid-treated group. Interestingly, this was not accompanied by an increase in short-term (plasma glucose) or long-term (blood HbA1c) blood glucose measurements.

**Table 1:** Total cholesterol and ALT in blood samples increased after topical dexamethasone treatment. Blood samples were analyzed after 4 weeks of treatment. All data are expressed as the mean ± standard deviation (*N* = 6)

Terms	Unit	Vehicle	DEX	*p* value
Albumin	(g/dL)	4.15 ± 0.12	4.05 ± 0.36	0.5359
ALP	(IU/L)	525.5 ± 74.9	656.8 ± 169	0.117
**ALT (GPT)**	**(IU/L)**	**57.0 ± 23.1**	**190.7 ± 169**	**0.0197**
AST (GOT)	(IU/L)	93.5 ± 23.4	164.7 ± 89.8	0.0897
Calcium	(mg/dL)	10.3 ± 0.32	10.1 ± 0.35	0.29
Chloride	(mEQ/L)	102.0 ± 1.91	101.0 ± 1.67	0.2487
Cholineesterase	(IU/L)	6.83 ± 2.04	5.50 ± 0.55	0.1533
**Creatinine**	**(mg/dL)**	**0.26 ± 0.03**	**0.20 ± 0.04**	**0.0132**
Free fatty acid	(*μ*EQ/L)	176.7 ± 80.0	382.7 ± 245.6	0.0793
Glucose	(mg/dL)	169.7 ± 13.2	166.2 ± 57.9	0.888
HbA1c (NGSP)	(%)	4.87 ± 0.37	5.55 ± 0.92	0.12234
Inorganic phosphorus	(mg/dL)	7.57 ± 0.56	8.30 ± 1.67	0.331
Potassium	(mEQ/L)	4.78 ± 0.50	4.82 ± 1.08	0.9466
Sodium	(mEQ/L)	143.8 ± 0.98	145.2 ± 1.60	0.1129
**Total cholesterol**	**(mg/dL)**	**70.0 ± 16.8**	**230.5 ± 37.6**	**<0.0001**
Total protein	(g/dL)	6.23 ± 0.32	6.35 ± 0.37	0.5739
Triglyceride	(mg/dL)	165.2 ± 63.3	208.2 ± 47.2	0.2119
Urea nitrogen	(mg/dL)	18.62 ± 1.56	19.28 ± 2.42	0.583
*γ* – GTP	(IU/L)	<3	<3	

*ALP* alkaline phosphatase, *ALT*, alanine transaminase, *GPT* glutamic pyruvic transaminase, *AST* aspartate aminotransferase, *GOT* glutamic oxaloacetic transaminase, *HbA1c* glycated hemoglobin, γ-*GTP* gamma-glutamyl transpeptidase

Bold data represent the term that have significantly difference between control and dex treatment

### RGC markers and ER stress markers were not altered by topical ocular dexamethasone

**Fig. 3:**
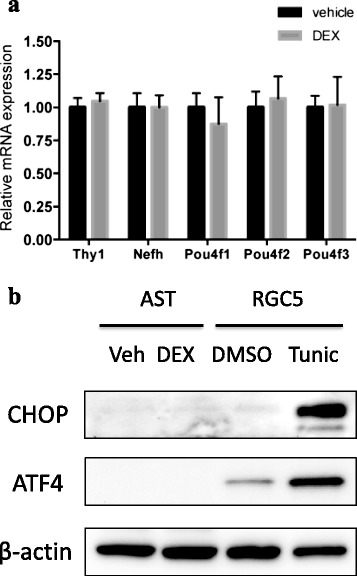
**a** Gene expression of RGC markers in the rat retinas was unaltered after topical dexamethasone treatment. The relative mRNA level of RGC markers was compared in the eyes of rats treated with 0.1 % dexamethasone (DEX) or vehicle for 4 weeks (each *N* = 5). The expression of the RGC markers was normalized to Gapdh. Values represent the mean ± standard deviation. **b** The expression of ER stress markers in the rat anterior segment was unaltered after topical dexamethasone treatment. Topical dexamethasone did not elevate the ER stress markers CHOP and ATF4 in the rats. Protein levels of CHOP and ATF4 in anterior segment tissue (AST) were examined with an immunoblot analysis in the rat eyes treated with saline vehicle (Veh) or 0.1 % dexamethasone (DEX) for 4 weeks. Tunicamycin (Tunic)-treated-RGC5 cells were used as a positive control for ER stress induction. DMSO was used as a vehicle for the tunicamycin treatment in the RGC5 cells. Beta-actin served as the loading control (*N* = 3)

After collecting the eyes at the 4-week time point, we quantified the gene expression of Thy1, Nefh, Pou4f1, Pou4f2 and Pou4f3, all regarded as constitutive RGC markers, to assess damage to the RGCs after the topical administration of dexamethasone (Fig. 3a). We found no significant difference in RGC marker expression between the eyes treated with topical steroid and those treated with saline. As the reduced expression of RGC markers precedes the loss of the RGCs themselves, these results were consistent with the failure to induce elevated IOP and the consequent RGC loss. In addition, we found that there was no obvious loss of the RGCs located in the GCL. Furthermore there was no detectable loss of other types of retinal cells nor were there any histological abnormalities (Additional file 1: Figure S2). Previous studies that used mice showed that the ER stress response was activated in the anterior segment early in the disease process, particularly in the trabecular meshwork, leading to IOP elevation [18]. To determine if this also occurred in rats after topical steroid treatment for 4 weeks, we tested for the induction of ER stress by analyzing the protein expression of the representative ER stress markers ATF4 and CHOP [21–23]. Both of these markers, previously shown to be elevated in dexamethasone-treated mouse eyes, were not up-regulated after topical steroid treatment in rats (Fig. 3b).

## Discussion

In this study, we initially attempted to replicate, in rats, a technique to induce IOP elevation that had been previously reported in mice [18]. However, to our surprise, we found that topical ocular dexamethasone administration resulted in a paradoxical decrease in IOP.

This decrease in IOP was preceded by a rapid reduction in body weight that was not described in the original findings in mice [18]. Assuming that body weight was indeed unaffected by dexamethasone treatment in mice, this difference may explain the IOP decrease we observed. The loss of body weight after corticosteroid use in rats was unexpected as it is known that common side effects of glucocorticoid in humans include gain of weight. However, the development of stomach irritation is also commonly observed, which could have led to the loss of appetite and weight. Another possibility is the taste alteration following corticosteroid use, which may have also reduced their appetite and body weight. We also observed increased cholesterol and ALT levels in the rats following treatment, but it is relatively unlikely that these biochemical parameters had a direct effect on IOP. Elevated ALT and total cholesterol are consistent with liver dysfunction as hepatocytes are involved in the metabolism of these molecules. However, it is difficult to link directly the liver dysfunction and the loss of weight; the association remains unclear. Similarly, a reduced IOP is not a common findings in patients with liver dysfuction, thus their relationship is also uncertain. A possible explanation for the reduced IOP includes the reduction of orbital fat and orbital pressure accompanied by the weight loss. Indeed, a recent report has shown that cardiometabolic risk factors, including total cholesterol, are associated with increased IOP in Korean subjects [24]. High levels of total cholesterol did not induce a similar elevation in IOP in the glucocorticoid-treated rats in our study, possibly due to differences in species and the period of increased total cholesterol. Additionally, elevated ALT in human subjects has not been shown to have a convincing association with increased IOP or glaucoma.

Moreover, as dexamethasone has been reported to increase mRNA and protein levels of ALT in mice [25], it is possible that ALT might be similarly affected in rats following treatment. In this case, the effect of dexamethasone on ALT levels would not account for the different IOP responses in the two different types of rodent. Another notable difference between this study and the previous work that used mice, which may have affected the systemic side effects of dexamethasone we observed, was the relatively lower dose given to the rats. Rats are 10 times heavier than mice, but the dose was only increased 2.5-fold. Finally, contradictory pharmacological responses in moderately related species are not unprecedented. For example, the intraperitoneal injection of dexamethasone induces hepatocellular necrosis in rats [26], but not in mice [25].

It is unclear why steroid treatment led to a decrease in body weight. As steroid treatment can induce diabetes, which may result in a reduction of body weight, we anticipated that blood glucose would be elevated as the result of topical ocular dexamethasone. However, a biochemical analysis of the blood samples showed that this straightforward outcome did not occur. In fact, only total cholesterol and ALT levels were elevated in the rats treated with topical dexamethasone. Regardless of the exact pathological mechanism, glucocorticoid treatment has been shown to be associated with reduced body weight [27]. As this treatment strongly promotes protein catabolism, it can also induce muscle atrophy as a side effect [28]. This is believed to be mediated by the sequential suppression of myostatin, the activation of mTOR, and the induction of the FoxO cascade, which results in promotion of autophagy of the muscles [29–31].

## Conclusions

In conclusion, topical ocular dexamethasone instillation resulted in decreased IOP in rats, a reaction that was opposite to that previously observed in mice. As this was accompanied by the loss of body weight and the elevation of plasma cholesterol and ALT, it appears that rats are more sensitive than mice to systemic side effects from ocular steroid treatment.

## Additional file

Additional file 1: Figure S1. IOP reduction in untreated left eye after topical dexamethasone treatment in rat. Topical ocular vehicle or DEX was administered 3 times daily for up to 4 weeks in the right eye. IOP measurements of untreated contralateral left eye from vehicle-treated and 0.1% dexamethasone-treated rats are shown. Values represent the mean ± standard deviation (*N* = 12). ^*^ *P* < 0.05, ^***^ *P* < 0.001, unpaired t-test. **Figure S2.** No alteration of the retinal histology or loss of RGCs observed following topical ocular treatment with dexamethasone in the rats. Retinal histological sections were stained by H&E. GCL, ganglion cell layer; IPL, inner plexiform layer; INL, inner nuclear layer; OPL, outer plexiform layer; ONL, outer nuclear layer. Scale bars=50 μm. (DOCX 119 kb)

## Abbreviations

ALT: Alanine transaminase
DEX: Dexamethasone
GCL: Ganglion cell layer
HbA1c: Glycated hemoglobin
IOP: Intraocular pressure
qRT-PCR: Quantitative reverse transcriptase polymerase chain reaction
RGCs: Retinal ganglion cells
SDS-PAGE: Sodium dodecyl sulfate-poly- acrylamide gel electrophoresis

## References

[1] Resnikoff S, Pascolini D, Etya'ale D, Kocur I, Pararajasegaram R, Pokharel GP, Mariotti SP (2004). Global data on visual impairment in the year 2002. Bull World Health Organ.

[2] Quigley HA (1996). Number of people with glaucoma worldwide. Br J Ophthalmol.

[3] Weinreb RN, Khaw PT (2004). Primary open-angle glaucoma. Lancet.

[4] Leske MC, Heijl A, Hussein M, Bengtsson B, Hyman L, Komaroff E, Early Manifest Glaucoma Trial G (2003). Factors for glaucoma progression and the effect of treatment: the early manifest glaucoma trial. Arch Ophthalmol.

[5] Beato M (1989). Gene regulation by steroid hormones. Cell.

[6] Losel R, Wehling M (2003). Nongenomic actions of steroid hormones. Nat Rev Mol Cell Biol.

[7] Beck RW, Cleary PA, Trobe JD, Kaufman DI, Kupersmith MJ, Paty DW, Brown CH (1993). The effect of corticosteroids for acute optic neuritis on the subsequent development of multiple sclerosis. The Optic Neuritis Study Group. N Engl J Med.

[8] Kirwan JR (1995). The effect of glucocorticoids on joint destruction in rheumatoid arthritis. The Arthritis and Rheumatism Council Low-Dose Glucocorticoid Study Group. N Engl J Med.

[9] Boumpas DT, Chrousos GP, Wilder RL, Cupps TR, Balow JE (1993). Glucocorticoid therapy for immune-mediated diseases: basic and clinical correlates. Ann Intern Med.

[10] Schacke H, Docke WD, Asadullah K (2002). Mechanisms involved in the side effects of glucocorticoids. Pharmacol Ther.

[11] Sholter DE, Armstrong PW (2000). Adverse effects of corticosteroids on the cardiovascular system. Can J Cardiol.

[12] Bernstein HN, Mills DW, Becker B (1963). Steroid-induced elevation of intraocular pressure. Arch Ophthalmol.

[13] Kersey JP, Broadway DC (2006). Corticosteroid-induced glaucoma: a review of the literature. Eye (Lond).

[14] Armaly MF, Becker B (1965). Intraocular pressure response to topical corticosteroids. Fed Proc.

[15] Becker B (1965). Intraocular pressure response to topical corticosteroids. Invest Ophthalmol.

[16] Clark AF, Wordinger RJ (2009). The role of steroids in outflow resistance. Exp Eye Res.

[17] Jones R, Rhee DJ (2006). Corticosteroid-induced ocular hypertension and glaucoma: a brief review and update of the literature. Curr Opin Ophthalmol.

[18] Zode GS, Sharma AB, Lin X, Searby CC, Bugge K, Kim GH, Clark AF, Sheffield VC (2014). Ocular-specific ER stress reduction rescues glaucoma in murine glucocorticoid-induced glaucoma. J Clin Invest.

[19] Li S, Lee J, Zhou Y, Gordon WC, Hill JM, Bazan NG, Miner JH, Jin M (2013). Fatty acid transport protein 4 (FATP4) prevents light-induced degeneration of cone and rod photoreceptors by inhibiting RPE65 isomerase. J Neurosci.

[20] Sato K, Li S, Gordon WC, He J, Liou GI, Hill JM, Travis GH, Bazan NG, Jin M (2013). Receptor interacting protein kinase-mediated necrosis contributes to cone and rod photoreceptor degeneration in the retina lacking interphotoreceptor retinoid-binding protein. J Neurosci.

[21] Zinszner H, Kuroda M, Wang X, Batchvarova N, Lightfoot RT, Remotti H, Stevens JL, Ron D (1998). CHOP is implicated in programmed cell death in response to impaired function of the endoplasmic reticulum. Genes Dev.

[22] Ohoka N, Yoshii S, Hattori T, Onozaki K, Hayashi H (2005). TRB3, a novel ER stress-inducible gene, is induced via ATF4-CHOP pathway and is involved in cell death. EMBO J.

[23] Oyadomari S, Mori M (2004). Roles of CHOP/GADD153 in endoplasmic reticulum stress. Cell Death Differ.

[24] Kim YH, Jung SW, Nam GE, Do Han K, Bok AR, Baek SJ, Cho KH, Choi YS, Kim SM, Ju SY, Kim DH (2014). High intraocular pressure is associated with cardiometabolic risk factors in South Korean men: Korean National Health and Nutrition Examination Survey, 2008–2010. Eye (Lond).

[25] Reagan WJ, Yang RZ, Park S, Goldstein R, Brees D, Gong DW (2012). Metabolic adaptive ALT isoenzyme response in livers of C57/BL6 mice treated with dexamethasone. Toxicol Pathol.

[26] Jackson ER, Kilroy C, Joslin DL, Schomaker SJ, Pruimboom-Brees I, Amacher DE (2008). The early effects of short-term dexamethasone administration on hepatic and serum alanine aminotransferase in the rat. Drug Chem Toxicol.

[27] De Vos P, Saladin R, Auwerx J, Staels B (1995). Induction of ob gene expression by corticosteroids is accompanied by body weight loss and reduced food intake. J Biol Chem.

[28] Schakman O, Gilson H, Kalista S, Thissen JP (2009). Mechanisms of muscle atrophy induced by glucocorticoids. Horm Res.

[29] Salehian B, Mahabadi V, Bilas J, Taylor WE, Ma K (2006). The effect of glutamine on prevention of glucocorticoid-induced skeletal muscle atrophy is associated with myostatin suppression. Metabolism.

[30] Shimizu N, Yoshikawa N, Ito N, Maruyama T, Suzuki Y, Takeda S, Nakae J, Tagata Y, Nishitani S, Takehana K (2011). Crosstalk between glucocorticoid receptor and nutritional sensor mTOR in skeletal muscle. Cell Metab.

[31] Sandri M, Sandri C, Gilbert A, Skurk C, Calabria E, Picard A, Walsh K, Schiaffino S, Lecker SH, Goldberg AL (2004). Foxo transcription factors induce the atrophy-related ubiquitin ligase atrogin-1 and cause skeletal muscle atrophy. Cell.

