# Supplementary material for: Topical ocular dexamethasone decreases intraocular pressure and body weight in rats
Source: J Negat Results Biomed. 2016 Mar 12;15:5. doi: 10.1186/s12952-016-0048-x (PMC4789283; doi:10.1186/s12952-016-0048-x)
Supplement: Additional file 1: Figure S1. — IOP reduction in untreated left eye after topical dexamethasone treatment in rat. Topical ocular vehicle or DEX was administered 3 times daily for up to 4 weeks in the right eye. IOP measurements of untreated contralateral left eye from vehicle-treated and 0.1% dexamethasone-treated rats are shown. Values represent the mean ± standard deviation (N = 12). * P < 0.05, *** P < 0.001, unpaired t-test. Figure S2. No alteration of the retinal histology or loss of RGCs observed following topical ocular treatment with dexamethasone in the rats. Retinal histological sections were stained by H&E. GCL, ganglion cell layer; IPL, inner plexiform layer; INL, inner nuclear layer; OPL, outer plexiform layer; ONL, outer nuclear layer. Scale bars=50 μm. (DOCX 119 kb) [file 12952_2016_48_MOESM1_ESM.docx]

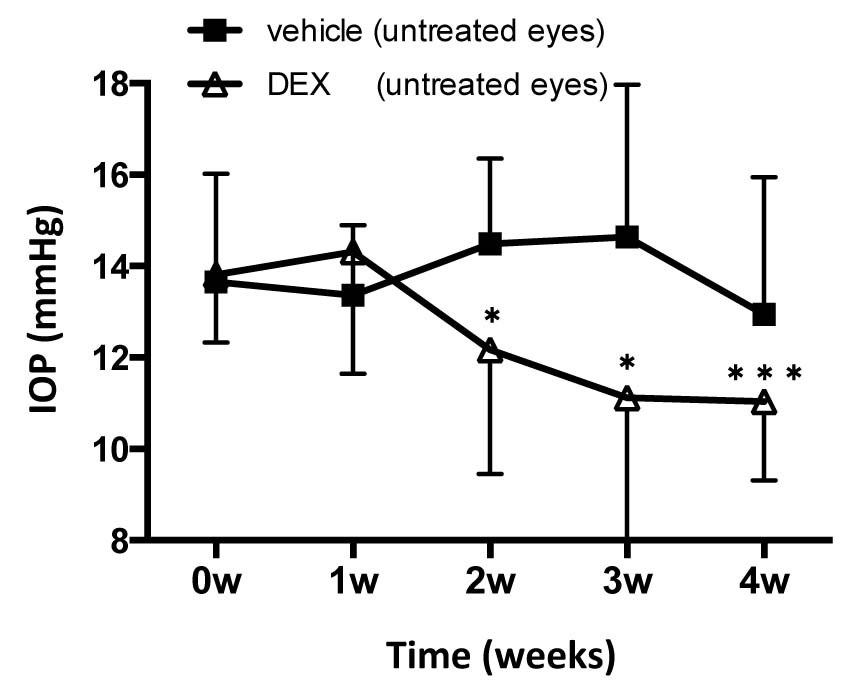


Figure S1.

IOP reduction in untreated left eye after topical dexamethasone treatment in rat. Topical ocular vehicle or DEX was administered 3 times daily for up to 4 weeks in the right eye. IOP measurements of untreated contralateral left eye from vehicle-treated and 0.1% dexamethasone-treated rats are shown. Values represent the mean ± standard deviation (N = 12). ^＊^P < 0.05, ^＊＊＊^P < 0.001, unpaired t-test.


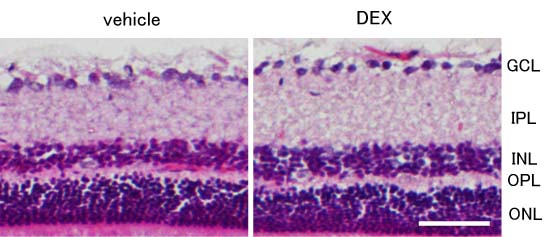


Figure S2.

No alteration of the retinal histology or loss of RGCs observed following topical ocular treatment with dexamethasone in the rats. Retinal histological sections were stained by H&E. GCL, ganglion cell layer; IPL, inner plexiform layer; INL, inner nuclear layer; OPL, outer plexiform layer; ONL, outer nuclear layer. Scale bars=50 μm.

<Methods>

*Cryosections and H&E staining*

Cryosections and H&E staining of rat retina were performed as described previously with minor modifications [1]. Hematoxylin staining was performed with hematoxylin solution (Type M, Muto Pure Chemicals, Tokyo, Japan) for 1 min, and sections were then stained for 30 s with 0.3% eosin alcohol solution (Muto Pure Chemicals). Images of stained rat retinal sections were captured with an Olympus BX53 microscope using a 10× objective lens and software (Standard Cellsens, Olympus, Tokyo, Japan).

<Reference>

1. Sato K, Ozaki T, Ishiguro S, Nakazawa M: **M-opsin protein degradation is inhibited by MG-132 in Rpe65(-)/(-) retinal explant culture.** *Mol Vis* 2012, **18:**1516-1525.
